# Effects of wearing masks during COVID-19 pandemic on the composition and diversity of skin bacteria and fungi in medical workers

**DOI:** 10.3389/fmicb.2023.1274050

**Published:** 2023-10-26

**Authors:** Jingxi Zhang, Peiyun Jiang, Yaxin Zhang, Wenlou Liu, Shujing Kong, Xiaoyang Hou, Zuoyao Qi, Yujin Sun, Guan Jiang

**Affiliations:** ^1^Department of Dermatology, The Affiliated Hospital of Xuzhou Medical University, Xuzhou, China; ^2^Xuzhou Medical University, Xuzhou, China; ^3^Department of Oncology, The Affiliated Hospital of Xuzhou Medical University, Xuzhou, China

**Keywords:** COVID-19 pandemic, masks, skin, metagenomic sequencing, microbial diversity

## Abstract

**Background:**

Although studies have shown that wearing masks can affect the skin microbiome, more detailed and comprehensive research on wearing masks needs to be further explored.

**Objective:**

This study aimed to characterize the influence of mask wearing on the diversity and structural characteristics of the facial skin microbial community of medical staff during the COVID-19 pandemic by means of metagenomic sequencing (mNGS).

**Methods:**

A total of 40 samples were taken by swabbing the cheek in the 2 × 2 cm^2^ area before and after wearing the masks. DNA was extracted for metagenomic sequencing.

**Results:**

A statistically significant decrease was found in the α diversity between BN and AN groups and between B2 h and A2 h groups. BN and AN mean groups before and after 8 h of wearing the medical protective mask (N95), including 10 volunteers, respectively. B2 h and A2 h mean groups before and after 8 h of wearing masks, including 10 volunteers changing mask every 2 h, respectively. The β diversity was found to be statistically reduced between BS and AS groups (*p* = 0.025), BN and AN groups (*p* = 0.009), and B2 h and A2 h group (*p* = 0.042). The fungal beta diversity was significantly decreased in every group before and after wearing masks. The main bacteria on the face before and after wearing masks were **Cutibacterium** (68.02 and 71.73%). Among the fungi, **Malassezia** predominated the facial skin surface before and after wearing masks (35.81 and 39.63%, respectively).

**Conclusion:**

Wearing different types of masks and changing masks according to different frequency will have different effects on the facial skin’s microbiota.

## Introduction

The skin is the biggest organ and an important barrier of the body to protect from external stimuli. Bacteria, fungi, viruses, and other microorganisms colonize its surface, maintaining dynamic homeostasis and forming a complex skin microecology. A healthy skin barrier is a rate-limiting layer through which various chemicals are absorbed to resist the entry of hazardous substances and irritants from the outside. It also has moisturizing and regulating function, performing a crucial physiological role in preserving the interior environment’s stability and resisting harmful external stimuli. One of the most frequent issues in dermatology is skin barrier degradation, and complex skin microecology is crucial for preserving the skin barrier.

The microbial community of healthy human skin surface has biogeographical differences, and the skin is divided into dry areas (such as forearm palmar side and thenar), wet areas (such as armpits, elbow fossa, and groin), and oily areas (such as forehead, nasal folds, and back) in accordance with its physiological state (Cogen et al., 2008).

Since the coronavirus disease 2019 (COVID-19) outbreak in Wuhan, China, in December 2019, severe acute respiratory syndrome coronavirus 2 (SARS-CoV-2) has caused a worldwide pandemic (Szegedi et al., 2019). It is highly infectious and spreads quickly, mostly by respiratory droplets and direct contact. SARS-CoV-2 carriers and confirmed cases can spread the virus to other people according to previous studies (Guan et al., 2020). Healthcare workers are joining the fight against COVID-19 in the face of sudden and enormous obstacles, and wearing masks is crucial to ensuring their safety at work. Although tight masks have strong antibacterial penetration capacity and could prevent the entry of harmful liquid substances, they make it difficult for perspiration to evaporate and keep medical workers’ skin wet. As a result, those wearing masks may have different microbial dispersion features on their face skin with normal environment. A number of skin issues, including pressure-related injuries, contact dermatitis, itching, stress urticaria, seborrheic dermatitis, and acne, have been linked to the use of masks (Szegedi et al., 2019). The comprehension of microbial diversity by using conventional culture methods is constrained by the difficult procedure and high demanding growth conditions (Stewart, 2012; Miquel et al., 2013). Metagenomic sequencing (mNGS), which first appeared in 2004, is an efficient technological tool that has expanded the possibilities for microbial research in the identification and categorization of microbes in clinical settings (Gu et al., 2019). By directly extracting all microbial DNA from the test sample, mNGS is able to identify and classify every present pathogen (Mathieu et al., 2014).

In this study, mNGS was used to assess the effects of wearing masks during the COVID-19 pandemic on the composition and diversity of skin bacteria and fungi in medical workers. Preventing and taking care of face skin injuries brought by wearing masks are meaningful for medical workers.

## Materials and methods

Twenty medical staff (ten males and ten females),with an average age of 24.45 ± 6.52 years (age range between 18 and 39 years) of the Affiliated Hospital of Xuzhou Medical University were enrolled into the present study. The healthcare workers enrolled in the study worked in environments with the same risk category. In accordance with the requirements, ten of them wore medical surgical masks (five males and five females) and the other ten wore medical protective masks (N95)(five males and five females) for a total of 8 h. Each group of 10 people was divided into half changing their masks every 2 h and the other half changing their masks every 4 h. However, we did not sample the skin during the mask replacement process. At 8:00 a.m. on October 21, 2022, before wearing a mask, 20 medical workers had their faces swabbed for skin samples, and then they wore their masks. Eight hours after, that is, at 4:00 p.m. of October 21, 2022, the skin sample was collected again at the same location where the sample was taken before wearing the mask.

The inclusion criteria were as follows: (1) no inflammatory manifestations of systemic skin (such as redness, swelling, heat, and pain) or other skin diseases; (2) no history of antibiotics, antifungal drugs, and glucocorticoids within 6 months; (3) did not use drugs at the sampling site within 24 h before sampling; (4) not accompanied by tumors or other serious diseases; and (5) did not use personal protective equipment other than masks on the face at the same time. All healthcare workers in this study participated voluntarily and signed informed consent forms.

### Sample collection

In a room around 26°C, skin samples were taken from the face of medical workers before and after wearing a mask. A sterile cotton swab soaked in sterile 0.9% NaCl (Shandong Hualu Pharmaceutical Co., Ltd.; China Xinxiang Huaxi Eisai Co., Ltd.) was used to collect samples from the 2 × 2 cm sampling point of the cheeks. The sampling method was as follows: a cotton swab was used to wipe the sampling area up and down 70 times for 45 s, sterile forceps was used to aseptically move the swab head into a 1.5 mL sterile EP tube, and the sample was stored at −80°C.

The samples were named as follows: the skin samples taken from the face before and after wearing the mask were named B and A, respectively. N means wearing an N95 mask, and S means wearing a general surgical mask. 2 h means changing mask every 2 h, and 4 h means changing mask every 4 h. However, we did not sample the skin during the mask replacement process. We sampled the same spot on the face only before and eight hours after wearing the mask, that is, twice in total.

### Sample processing and nucleic acid extraction

The collected skin swab sample was frozen immediately and transported to the laboratory for mNGS testing. The experimental procedure of mNGS included DNA extraction, DNA quality detection, library construction, library purification, library quality inspection, library quantification, and sequencing. Firstly, the samples were extracted using the Novizan FastPure Microbiome DNA Isolation Kit (item No.: DC502). Secondly, Qubit tests DNA concentration. And then, DNA sample construction was performed using Novizan Universal Plus DNA Library Prep Kit for Illumina (item No. ND617). After the library is constructed, we use Qubit for initial quantification and dilute the library to 2 ng/ul. Then we use Fragment Analyzer to detect the insert size of the library. After the insert size meets the expectation, Q-PCR method is used to accurately quantify the effective concentration of the library (effective concentration of the library >3 M) to ensure the quality of the library.

Each step of the experimental process was strictly quality controlled. After passing the test, different libraries were mixed in accordance with the requirements of effective concentration and target data volume under the machine. Afterwards, Illumina PE150 sequencing was performed.

### Bioinformatics analysis

Raw data were obtained by sequencing on the Illumina sequencing platform (Kneaddat, Bowtie2). Quality control and host filtering were conducted to obtain effective data (Clean Data). The sequence alignment software HiSAT2 (version 2.0.1) was used to compare the high-quality microbial data in the universal microbial database, and the successfully matched microbial genomes were annotated by species. The relative abundance of microorganisms was calculated by RPKM standardization. Mothur software was used to calculate the Shannon and Simpson indices to assess microbial α diversity. The higher the value of these indices was, the higher the community diversity. R software (version 3.4.3) was used for principal coordinate analysis (PCoA) on the basis of weighted bray_curtis distance to assess β diversity by correlation and significant differences in species composition between microbial communities before and after wearing a mask.

### Statistical methods

The data obtained in this study were statistically analyzed using SPSS26.0 and R. Paired t-test was conducted to compare the α diversity between two groups. ANOSIM was used to analyze whether a statistically significant difference existed in the β diversity between two groups, and *R* > 0 indicated a difference between groups. Moreover, statistically significant difference was set to *p* < 0.05. The abundance of genus before and after wearing masks was compared by paired sample t-test, and the statistically significant difference was both set at *p* < 0.05.

## Results

### Sequencing results

A total of 40 skin samples were obtained from 20 adult volunteers. The total numbers of reads for each single sample before and after wearing a mask were from 40.11 to 68.35 M and from 42.88 to 68.63 M, respectively. The numbers of high-quality reads in a single sample before and after wearing a mask were 1.34–30.46 and 1.258–29.40 M, respectively, accounting for 2.76–54.84% and 2.56–54.95% of the total number of sequenced reads, respectively.

### Changes in microbial community diversity before and after wearing a mask

#### Analysis of α diversity index of microbial communities

**Figure 1:**
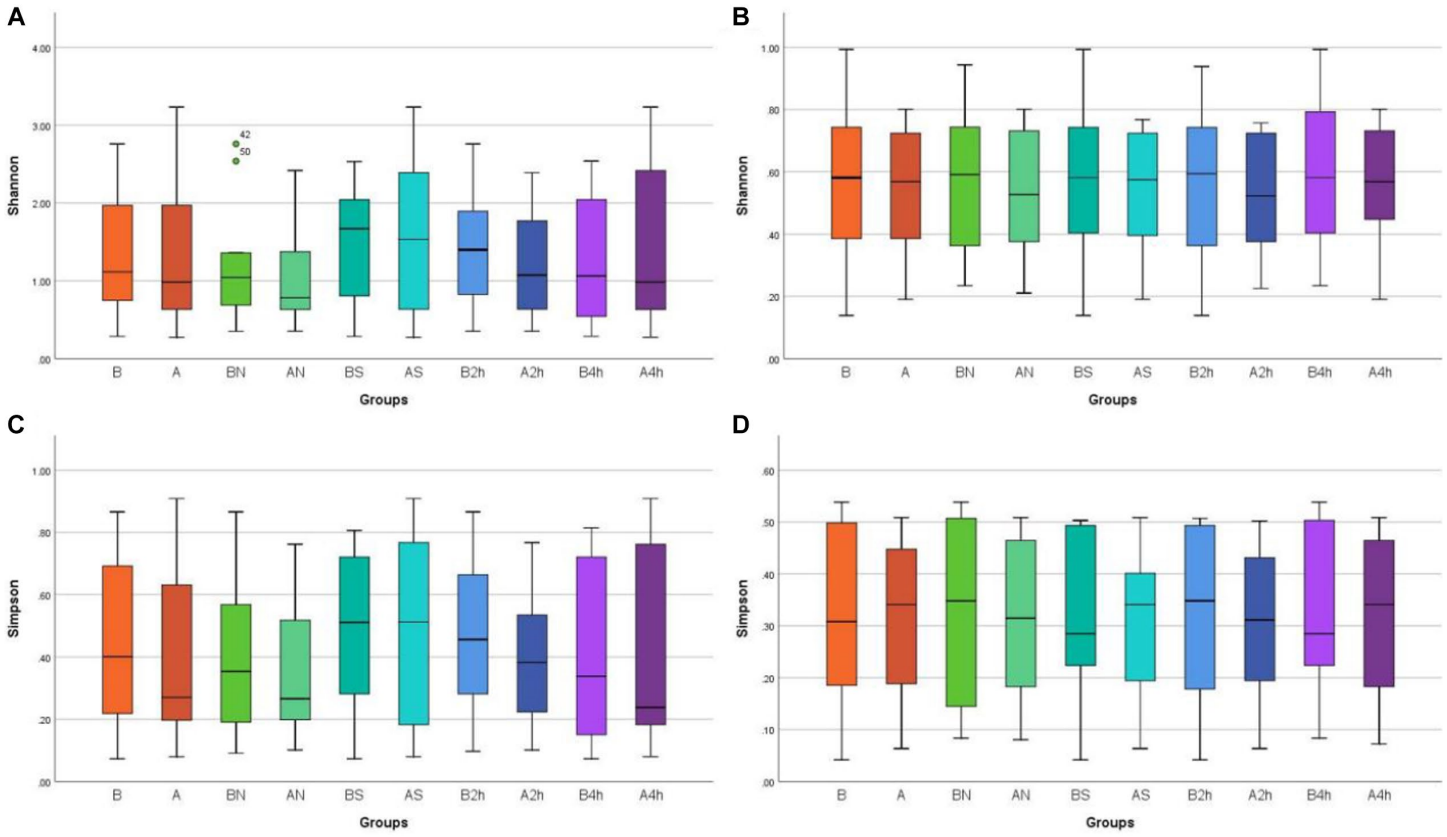
Box plots of α diversity differences in different mask groups and different time groups **(A)** Bacteria Shannon **(B)** Fungi Shannon **(C)** Bacteria Simpson **(D)** Fungi Simpson.

Shannon and Simpson indices were used to assess the α diversity of each group and changes in microbial diversity among individuals. The bacterial Shannon and Simpson indices before and after wearing a mask were as follows: compared with the B group, the A group showed no significant difference in both indices (*p* > 0.05). No significant difference was also found between the Shannon index in the AN group and the A2 h group compared with that of the BN group and the B2 h group (*p* > 0.05), but the Simpson index was statistically different (*p* < 0.05). No significant difference was found between the Shannon and Simpson indices in the AS and A4 h group compared with those in the BS and B2 h groups (*p* > 0.05, Tables 1, 2). The α diversity difference between groups was visualized using box plots (Figures 1A,B).

**Table 1:** Comparison of bacterial Shannon index (x ± s) in skin samples before and after wearing masks.

Group	Total sample	N95 mask group	Surgical mask group	2 h change group	4 h change group
Before	1.35 ± 0.18	1.21 ± 0.26	1.5 ± 0.25	1.46 ± 0.24	1.25 ± 0.27
After	1.3 ± 0.20	1.07 ± 0.22	1.53 ± 0.33	1.26 ± 0.23	1.35 ± 0.34
*T* value	−0.195	−0.401	0.078	−0.623	0.236
*p* value	0.846	0.694	0.939	0.541	0.816

**Table 2:** Comparison of bacterial Simpson index (x ± s) of skin samples before and after wearing masks.

Group	Total sample	N95 mask group	Surgical mask group	2 h change group	4 h change group
Before	0.44 ± 0.26	0.48 ± 0.23	0.41 ± 0.28	0.47 ± 0.23	0.42 ± 0.28
After	0.41 ± 0.26	0.41 ± 0.22	0.41 ± 0.30	0.40 ± 0.23	0.42 ± 0.29
*T* value	1.575	2.650	−0.056	2.987	−0.156
*p* value	0.132	0.026	0.957	0.015	0.872

The changes in the Shannon and Simpson indices of fungi before and after wearing a mask were as follows: no significant difference was observed in the B, BN, BS, B2h, and B4 h groups compared with those in the A, AN, AS, A2h, and A4 h groups (all p > 0.05), as shown in Tables 3, 4. The α diversity difference between groups was visualized using box plots (Figures 1C,D).

**Table 3:** Comparison of fungal Shannon index (x ± s) of skin samples before and after wearing masks.

Group	Total sample	N95 mask group	Surgical mask group	2 h change group	4 h change group
Before	0.58 ± 0.05	0.57 ± 0.07	0.6 ± 0.08	0.56 ± 0.08	0.61 ± 0.08
After	0.53 ± 0.05	0.53 ± 0.06	0.53 ± 0.06	0.52 ± 0.06	0.54 ± 0.07
*T* value	−0.74072	−0.39114	−0.62392	−0.36668	−0.65655
*p* value	0.4636	0.7003	0.541	0.7183	0.5199

**Table 4:** Comparison of fungal Simpson index (x ± s) of skin samples before and after wearing masks.

Group	Total sample	N95 mask group	Surgical mask group	2 h change group	4 h change group
Before	0. 33 ± 0. 16	0.32 ± 0. 17	0.33 ± 0. 15	0.37 ± 0. 12	0.28 ± 0. 18
After	0.31 ± 0. 15	0.30 ± 0. 15	0.32 ± 0. 16	0.37 ± 0. 11	0.25 ± 0. 16
*T* value	1.061	1.067	0.474	0.131	1.383
*p* value	0.302	0.314	0.647	0.898	0.200

#### Analysis of β diversity index of microbial communities

**Figure 2:**
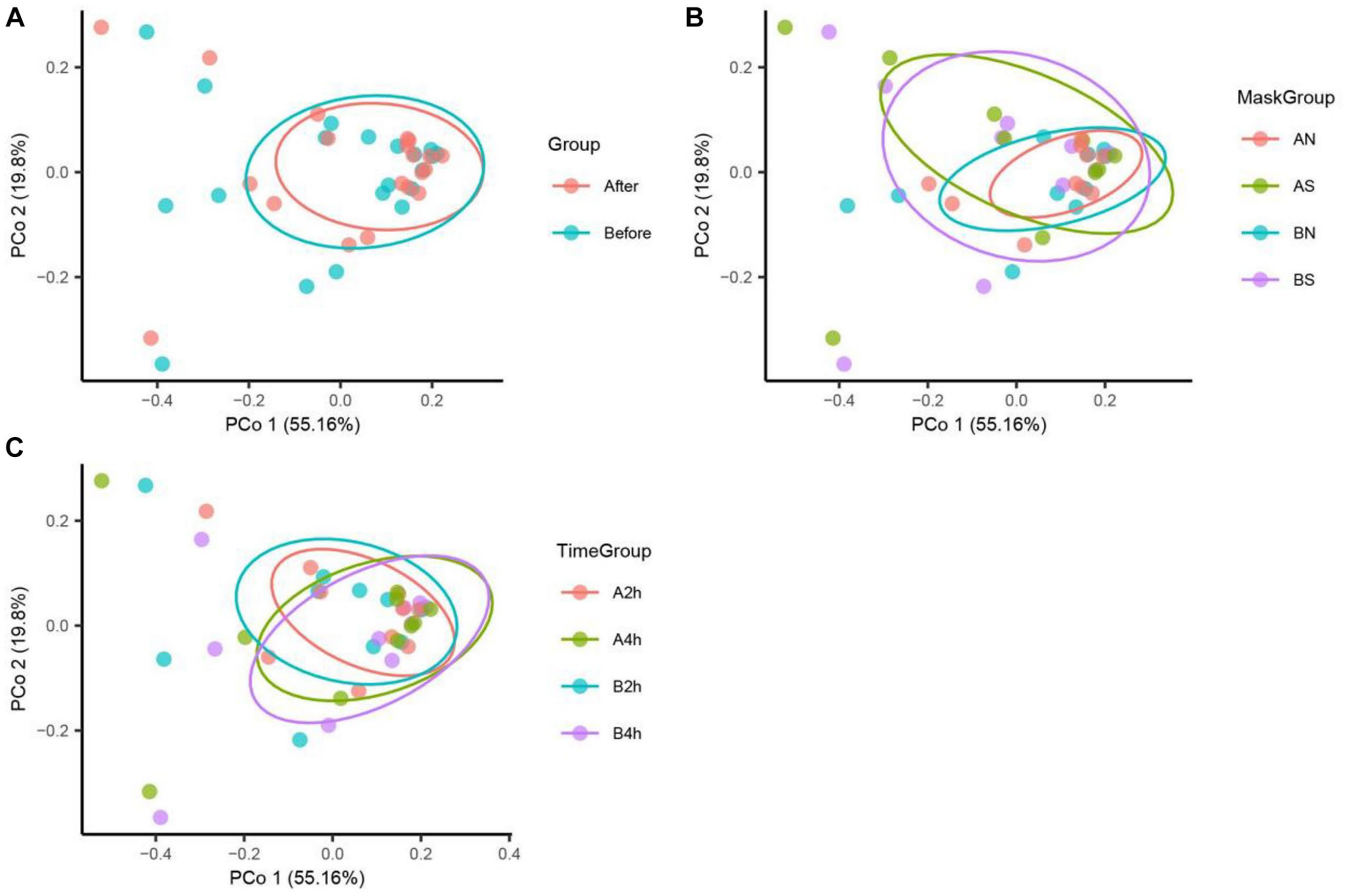
Bacteria PCoA analysis in different mask groups and time groups. **(A)** Total Group, **(B)** Mask Group, **(C)** Time Group.

**Figure 3:**
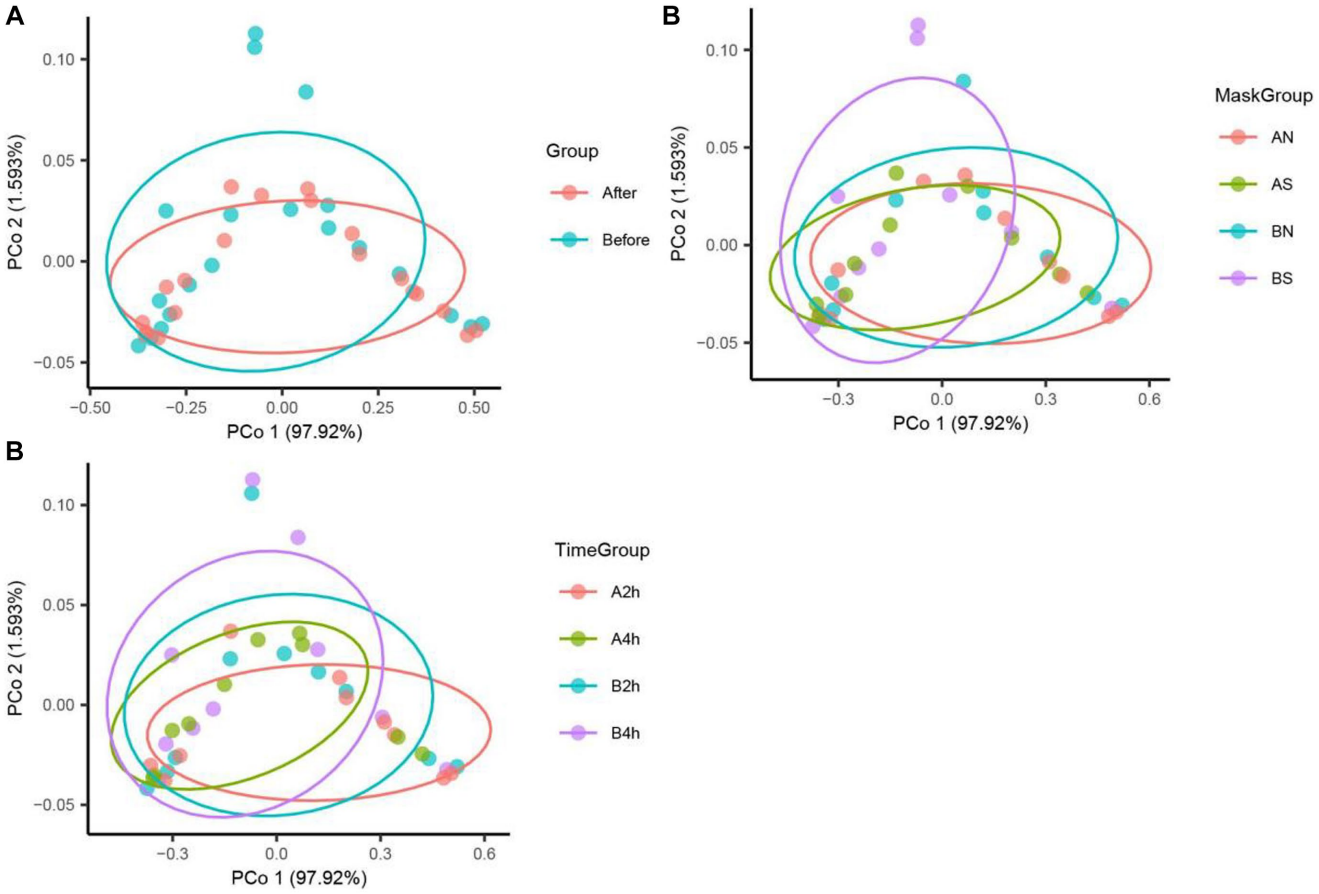
Fungi PCoA analysis in different mask groups and time groups. **(A)** Total Group, **(B)** Mask Group, **(C)** Time Group.

The PCoA of unweighted bray_curtis distance was conducted to study the changing trend of microorganisms in different groups after wearing a mask. In the samples before wearing a mask, the points of different individuals were scattered in the PCoA figure, indicating that the microbiota structure of different individuals was relatively different. The β diversity of fungal communities decreased significantly from the B, BN, BS, and B2 h groups to the A, AN, AS, and A2 h groups (*R* > 0, *p* < 0.05), whereas the B4 h and A4 h groups was not statistically different (*p* > 0.05). A significant decline was observed in the β diversity of fungal communities in every group between before and after wearing a mask (all R > 0, p < 0.05, Table 5). The PCoA figures are shown in Figures 2, 3.

**Table 5:** Analysis of the β diversity of bacterial and fungal communities (β diversity) at the genus level before and after wearing the mask.

	*R* value (bacteria)	*p* value (bacteria)	*R* value (fungi)	*p* value (fungi)
B-A	0.07039	0.025	0.3228	0.001
BN-AN	0.1563	0.009	0.2201	0.035
BS-AS	0.1822	0.025	0.4057	0.002
B2 h-A2h	0.1038	0.042	0.4986	0.001
B4 h-A4h	0.05433	0.125	0.2133	0.019

### Microbial composition analysis

#### Analysis of bacterial composition before and after wearing a mask

**Figure 4:**
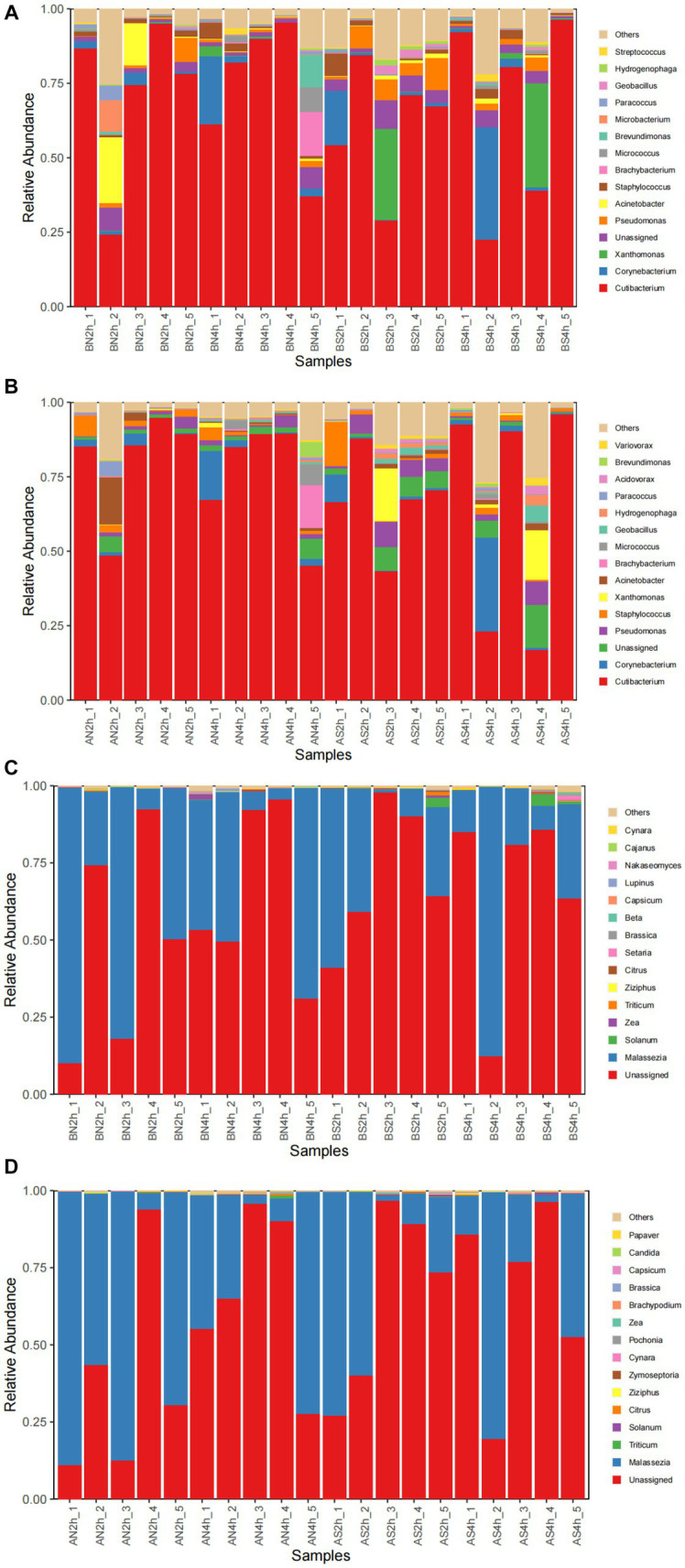
**(A)** The relative abundance of the top 15 bacterial genera before wearing masks; **(B)** The relative abundance of top 15 bacterial genera after wearing masks; **(C)** The relative abundance of the top 15 fungal genera before wearing masks; **(D)** The relative abundance of top15 fungal genera after wearing masks.

The bacterial composition of the samples collected from facial skin surfaces were analyzed at the genus level. The relative abundance of the top 15 bacterial genus in the total sample is visualized in Figures 4A,B. Six bacterial genera had an abundance of in the total sample of facial skin before and after wearing a mask, with **Cutibacterium** as the main genus, with abundances of 68.02 and 71.73%, respectively. The A group had a higher relative abundance of **Cutibacterium** than the B group, without statistical significance (*p* = 0.09). The bacterial genera with a total sample abundance greater than 1% in both groups of skin are shown in Table 6. After wearing a mask, the abundance of **Corynebacterium** genus was lower than that of the group before wearing a mask, with statistically significant difference (*t* = −2.111, *p* < 0.05). Meanwhile, no statistically significant difference was observed in the abundance of other bacterial genera (*p* > 0.05).

**Table 6:** Bacterial genera with >1% abundance in the total facial skin samples before and after wearing the mask.

Before wearing a mask (20 cases)		After wearing a mask (20 cases)	
Bacterial genus	Abundance	Bacterial genus	Abundance
**Cutibacterium** (G +)	68. 02%	**Cutibacterium** (G +)	71.73%
**Corynebacterium** (G+)	5. 07%	**Corynebacterium** (G+)	3.83%
**Xanthomonas**	3. 63%	**Xanthomonas**	1. 93%
**Pseudomonas**	2. 76%	**Pseudomonas**	2. 60%
**Acinetobacter**	2. 27%	**Acinetobacter**	1. 45%
**Staphylococcus**	1. 79%	**Staphylococcus**	2. 36%

There was nine and seven bacterial genera having an abundance of >1% before and after wearing N95 masks respectively; both five before and after wearing surgical masks; seven and six before and after 2-h mask replacement, and eight and six before and after 4-h mask replacement. The bacterial genera with abundances over 1% in different groups of skin samples are shown in Table 7. No significant difference was found in the abundance of all the >1% bacterial genera in the surgical mask group and the 4-h replacement group (all *p* > 0.05). The abundance of **Cutibacterium** in the A2 h group was significantly higher than that in the B2 h group (*t* = 2.726, *p* < 0.05), and a certain increase was found from the BN group to the AN group without significant significance (*p* = 0.07 > 0.05). The independent sample *t*-test results showed no significant difference in the values of each bacterium between the N95 mask group and the surgical mask group (*p* > 0.05). On the contrary, a significant difference was found between the **Staphylococcus** genus in the 2 h and 4 h groups (*t* = −2.125, *p* = 0.048 < 0.05), indicating that changing masks every 2 h produced more **Staphylococcus** genus than every 4 h.

**Table 7:** Bacterial genera with >1% abundance in the total facial skin samples in all groups.

Before wearing a mask (10 cases)		After wearing a mask (10 cases)	
Bacterial genus	Abundance	Bacterial genus	Abundance
**N95 mask group**			
**Cutibacterium**	72. 42%	**Cutibacterium**	78. 00%
**Acinetobacter**	3. 87%	**Corynebacterium**	2. 91%
**Corynebacterium**	3. 66%	**Staphylococcus**	2. 19%
**Staphylococcus**	1. 61%	**Acinetobacter**	2. 11%
**Brachybacterium**	1. 57%	**Brachybacterium**	1. 511%
**Pseudomonas**	1. 56%	**Pseudomonas**	1. 510%
**Brevundimonas**	1. 29%	**Micrococcus**	1. 09%
**Micrococcus**	1. 18%		
**Microbacterium**	1. 12%		
**Surgical mask group**			
**Cutibacterium**	63. 62%	**Cutibacterium**	65. 45%
**Xanthomonas**	6. 81%	**Corynebacterium**	4. 74%
**Corynebacterium**	6. 48%	**Pseudomonas**	3. 68%
**Pseudomonas**	3. 96%	**Xanthomonas**	3. 63%
**Staphylococcus**	1. 98%	**Staphylococcus**	2. 53%
**2 h replacement group**			
**Cutibacterium**	66. 44%	**Cutibacterium**	73. 91%
**Pseudomonas**	4. 12%	**Pseudomonas**	3. 33%
**Acinetobacter**	4. 10%	**Staphylococcus**	3. 28%
**Xanthomonas**	3. 15%	**Acinetobacter**	2. 28%
**Corynebacterium**	3. 02%	**Corynebacterium**	1. 97%
**Staphylococcus**	1. 77%	**Xanthomonas**	1. 83%
**Microbacterium**	1. 11%		
**4 h replacement group**			
**Cutibacterium**	69. 60%	**Cutibacterium**	69. 54%
**Corynebacterium**	7. 13%	**Corynebacterium**	5. 68%
**Xanthomonas**	4. 12%	**Xanthomonas**	2. 04%
**Staphylococcus**	1. 81%	**Pseudomonas**	1. 86%
**Brachybacterium**	1. 58%	**Staphylococcus**	1. 44%
**Pseudomonas**	1. 40%	**Micrococcus**	1. 27%
**Micrococcus**	1. 31%		
**Brevundimonas**	1. 29%		

In the species level, this study focused on species with high abundance and potential harm, namely, *Staphylococcus aureus*, *Staphylococcus epidermidis*, *Pseudomonas aeruginosa*, *Corynebacterium jetnieri*, Corynebacterium, and *Propionibacterium acnes*. The analysis of their abundance demonstrated no significant differences among *S. aureus*, *S. epidermidis*, *P. aeruginosa*, and *C. jetnieri* before and after wearing a mask in all groups (*p* > 0.05). The relative abundance of *C. diphtheria* in the A group decreased compared with that in the B group but without significant significance (*p* = 0.1 > 0.05). The remaining groups also had no statistically significant difference. The abundance of *P. acnes* in the AN group was higher than that in the BN group (*p* = 0.09 > 0.05). The abundance of *P. acnes* in the A2 h group increased significantly compared with that in the B2 h group (t = −2.50, *p* < 0.05).

#### Analysis of fungal composition before and after wearing a mask

The top 15 fungal genera are shown in Figures 4C,D. The fungal genus in the total facial skin samples in each group was only **Malassezia**, with abundances of 35.63 and 39.81% before and after wearing a mask (*p* > 0.05).

**Malassezia** dominated the fungal genera in all the groups. Its relative abundance before and after wearing a mask in each group is shown in Table 6. No significant difference was found among the AN, AS, and A4 h groups and among the BN, BS, and B4 h groups (all *p* > 0.05), whereas the abundance of **Malassezia** in the A2 h group was significantly higher than that in the B2 h group (*t* = 2.274, *p* = 0.049 < 0.05). Meanwhile, the results of independent sample *t*-test exhibited no statistically significant difference in the abundance of fungal genera between the N95 mask group and the surgical mask group (*p* > 0.05) and between the 2 h replacement group and the 4 h replacement group (*p* > 0.05) (Table 8).

**Table 8:** Relative abundance of **Malassezia** in each group.

Group	Total sample	N95 mask group	Surgical mask group	2 h change group	4 h change group
Before	35. 63%	41. 85%	29. 42%	38. 76%	32.50%
After	39. 81%	46. 49%	33. 14%	47. 39%	32. 24%

## Discussion

Early understanding of microorganisms relied on traditional culture methods, but many microorganisms require special growth conditions, which limit the understanding of microbial diversity (Miquel et al., 2013). In recent years, the emergence of metagenomics has opened the door to microbial research, which directly extracts the genomic DNA of all microorganisms from environmental samples, and combines the PCR amplification and sequencing of genetic marker genes for research purpose to obtain species identification results. Many researchers use this method to study the community characteristics of the skin microbiota, which could be greatly affected by the surrounding environment. During the epidemic, the environment in which medical workers wear masks is different from that of ordinary wards, and medical workers are under great psychological pressure. This situation may result in a decrease in the diversity of the microbial diversity and an increase in the proportion of pathogenic or conditionally pathogenic bacteria.

The location of the sample in the present study was the cheek, which belongs to oily areas (NISC Comparative Sequencing Program et al., 2014). Moreover, healthcare workers were considered healthy before wearing masks. The main genus of the healthy human oily area is **Cutibacterium**. In the present study, the main bacterium of the skin sample was also **Cutibacterium**, with a relative abundance of 68.02%, followed by **Corynebacterium** at 5.07%, **Pseudomonas** species at 3.63%, **Xanthomonas** species at 3.32%, and **Staphylococcus** species at 2.76%, consistent with the results of previous studies (Lin et al., 2022; Perugini et al., 2023). In terms of fungi, all parts of the healthy human body are dominated by **Malassezia**. In the present study, the sequencing results found that **Malassezia** was the main fungus, having a relative abundance of 35.63%, which was lower than that of previous studies at 92.94% (Lin et al., 2022). The main reason for the difference in the experimental results is the heterogeneity of bacteria between individuals (Gao et al., 2007; Fierer et al., 2008; SanMiguel and Grice, 2015). In a study of the bacterial diversity of hands, researchers found that although there was a core set of bacterial taxa commonly found on the palm surface, they observed pronounced interpersonal variation in bacterial community composition: hands from different individuals sharing only 13%. Community composition was significantly affected by handedness, time since last hand washing, and an individual’s sex, with women having significant higher diversity (Fierer et al., 2008). The variation between individuals in microbial ecology was illustrated by this study.

During the pandemic, medical workers need to wear masks to protect the human body from harmful substances in the environment. When wearing masks, the evaporation of sweat on the surface of the skin is limited, and the heat generated by the human body is difficult to effectively dissipate, creating a relatively closed humid and hot microenvironment. Along with the extreme psychological pressure of medical workers, this condition may lead to a decrease in the microbial diversity and an increase in the proportion of pathogenic bacteria or conditional pathogens (Scarano et al., 2020). The present study explored the changes in human facial symbiotic microorganisms after wearing a mask, and the results showed that the bacterial α diversity of the AN and A2 h groups decreased dramatically compared with that of the BN and B2 h groups. Meanwhile, the bacterial β diversity of the A, AN, AS, and A2 h groups reduced statistically compared with that of the B, BN, BS, and B2 h groups. The fungal β diversity declined in every group after wearing a mask. Overall, the diversity of the skin surface microbiota decreased after wearing a mask, especially for the N95 and 2 h groups. In conclusion, the results of the present study indicate that the microbial diversity decreases due to skin environment changes after wearing a mask which is consistent with previous researches. The decline in the diversity of microbial communities is an important risk factor for the development of skin diseases (Cogen et al., 2008). The more obvious difference in the N95 group than in the surgical-mask group may be attributed to the N95 mask having poorer air permeability, and sweat is difficult to volatilize after long-time wearing masks. With more humid exhaled gas, the skin is in a hot and humid state for a long time, which may easily lead to whitening, softening, and wrinkling of the skin. As a result, the face is overhydrated and pH has changed, leading to a greater weakening of the skin’s protective barrier than that in the surgical-mask group (Minematsu et al., 2011; Young, 2018). The reasons for the more obvious difference in the 2 h group than in the 4 h group may be that the mask is replaced too frequently and the new mask is harder, making the mechanical friction destroy the skin’s protective barrier. Therefore, changing masks too often could also lead to an imbalance in the skin flora.

The level of sebum secretion on the face is more vigorous than before, and the pH is reduced after wearing a mask, which is conducive to the reproduction of **Cutibacterium** species. The results of this study demonstrated that the abundance of **Cutibacterium** in the N95 and 2 h groups was higher than before wearing a mask. Furthermore, the skin is in a relatively closed environment after wearing a mask, so the surface is moister and more anaerobic, which are conducive to the reproduction of anaerobic bacteria. In this study, the abundance of **Cutibacterium** in the N95 and 2 h groups increased after wearing a mask, and the total sample of **Corynebacterium** decreased significantly, may be due to the fact that **Cutibacterium** is an anaerobic bacterium and **Corynebacterium** is a facultative anaerobe (Cogen et al., 2008).

In recent years, many studies have reported adverse effects associated with healthcare workers’ wearing masks. A survey from Singapore showed that during the SARS pandemic, the incidence of adverse skin reactions due to mask wearing was 35.5%, with the most common adverse skin reactions being seat sores (59.6%), itching (51.4%), and pimples (35.8%) (Darlenski and Tsankov, 2020). Foo et al. reported that the most common adverse skin reactions after wearing N95 masks are acne, facial itching, and rash (Foo et al., 2006). Itching was speculated to be related to irritating contact dermatitis caused by mask components, such as adhesives, rubber in lacing, free formaldehyde released from nonwoven polypropylene materials, and metal in plastic nose strips (Foo et al., 2006; Hua et al., 2020). Moreover, the internal environment could be too humid due to wearing a mask for a long time,which may cause discomfort and dysbiosis. In the present study, the abundance of ***P. acnes*** increased significantly in the N95 and 2 h groups after wearing a mask. However, new findings on ***P. acnes*** suggested that contrary to previous beliefs, its proliferation is not a trigger for acne but an imbalance between members of the skin flora and between ***P. acnes*** phylotypes, which may play a more critical role in acne episodes (Tomida et al., 2013; Barnard et al., 2016; Szabó et al., 2017). In the present study, the species diversity of the microbiota decreased significantly after wearing a mask, and this finding was closely related to the onset of acne. In addition, Barnard suggested that some strains of ***P. acnes*** are highly associated with acne, whereas others are associated with healthy skin. Therefore, the balance between the phylotypes of ***P. acnes*** may also be disrupted. Meanwhile, local compression leads to obstruction of the lumen secretion of sebaceous glands in the hair follicles, which, in turn, leads to occlusion of the sebaceous ducts of the hair follicles to form a pimple (Kistowska et al., 2015; Tetzlaff et al., 2015). The great psychological pressure among medical staff is also an important factor in acne. When the human body is in a state of high mental tension for a long time, a strong stress response may lead to endocrine target gland axis secretion disorders, turning androgens into a highly active secretion state and stimulating a large amount of secretion by sebaceous glands to cause stress acne.

After wearing a mask, the fungi of the skin surface and the relative abundance of **Malassezia** did not significantly change, possibly because the growth cycle of fungi was longer than that of bacteria, and fungi were more tolerant to changes in skin physiological state than bacteria (Lin et al., 2022). The variation before and after wearing a mask in microbial ecology illustrated by this study emphasizes the challenges inherent in taking effective precautions when wearing a mask; addressing this challenge will be critical for the facial health of healthcare workers.

This study has limitations. (1) The possible presence of microbial contamination during sampling and laboratory operation and the difference in sampling intensity may be factors that affected the experimental results. (2) The time of wearing masks and the number of people in this study are limited. Large samples of individual wearing a mask for a long time should be collected for research in the future.

## Data availability statement

The datasets presented in this study can be found in online repositories. The names of the repository/repositories and accession number(s) can be found in the article/supplementary material.

## Ethics statement

The studies involving humans were approved by the Ethics Committee of Affiliated Hospital of Xuzhou Medical University. The studies were conducted in accordance with the local legislation and institutional requirements. The participants provided their written informed consent to participate in this study.

## Author contributions

JZ: Data curation, Formal analysis, Methodology, Writing – original draft. PJ: Data curation, Formal analysis, Writing – original draft. YZ: Data curation, Methodology, Writing – original draft. WL: Methodology, Writing – review & editing. SK: Data curation, Writing – original draft. XH: Methodology, Writing – original draft. ZQ: Writing – original draft. YS: Writing – original draft. GJ: Conceptualization, Methodology, Project administration, Resources, Supervision, Writing – review & editing.
